# General Concepts in Adult Congenital Heart Disease

**DOI:** 10.4274/balkanmedj.2017.0910

**Published:** 2018-01-20

**Authors:** Ferit Onur Mutluer, Alpay Çeliker

**Affiliations:** 1 Department of Cardiovascular Diseases, Koç University Hospital, İstanbul, Turkey; 2 Clinic of Pediatric Cardiology, American Hospital, İstanbul, Turkey

**Keywords:** Adult, congenital heart disease, general, concepts, special

## Abstract

Congenital heart disease in adults (adult congenital heart disease) is a growing burden for healthcare systems. While infant mortality due to congenital heart disease in the last four decades decreased by almost 3-fold, adult congenital heart disease prevalence increased by more than 2-fold in United States. Adult congenital heart disease prevalence is expected to increase steadily until 2050 in projections. Adult congenital heart disease is a multifaceted problem with many dimensions. This manuscript aims to provide an overview of the common adult congenital heart diseases and summarize important points in management of these diseases with possible problems and complications that the patients and the physicians face.

Congenital heart disease in adults [adult congenital heart disease: (ACHD)] is a growing burden for healthcare systems. The definition is the persistence of any structural abnormality present at birth that involves the heart and/or great vessels in adult life i.e. beyond 16 years of age. Arrhythmia and cardiomyopathies are not covered in this definition (1). Epidemiology of ACHD in our country is not clear. There is an estimated incidence of 8/1000 live births for congenital heart disease and thanks to advances in surgery and interventional cardiology, more than 85% of these patients live into adulthood today. There are approximately 50 million ACHD patients world-wide. ACHD prevalence is expected to increase steadily until 2050 in projections (Figure 1) (2). Congenital heart disease encompasses a wide variety of structural heart diseases and great vessels present at birth. Historically most of the patients bearing congenital heart defects had considerably decreased survival. Majority of the conditions were diagnosed at post-mortem examinations (3). Developments in drugs for the heart, surgery and interventional procedures, racing with each other, altered survival as well as natural progression of these diseases. Organization of cardiology practice as pediatric and adult cardiologists, and limited communication between these two specialist groups, resulted in increasing number of patients struggling to find optimal care for their conditions as they reach adulthood and admit to hospital with complex problems (4). Focus in modern healthcare system organization schemes is on ischemic heart disease and heart failure. Distribution of cases among high number of centers hamper the process of training certified experts in the field (5). Healthcare systems are in need of sufficient number of ACHD specialists, as well as specific workflows designed to connect patients scattered throughout large geographical areas to low number of ACHD specialists. Many ACHD patients have altered cardiovascular physiology due to a correction or an uncorrected complex defect. These patients might respond in an unexpected way to conventional treatments. A patient looking healthy might be exhibiting alarming findings that could only be visible to a trained eye. ACHD is a multi-faceted problem, and our aim in this manuscript is to provide an insight to the different dimensions of this problem. Featured sections are: Selected imaging modalities and recent developments in these modalities, ACHD commonly discovered in adults, issues related to selected ACHD groups with previous palliative or corrective surgeries and special considerations in ACHD patient population. A concise discussion of diseases is beyond the scope of this text and the readers are directed to relevant guidelines and textbooks in the field (6,7).

## IMAGING MODALITIES AND RECENT DEVELOPMENTS

### Echocardiography methods

Two-dimensional echocardiography (2D-TTE) is the mainstay of diagnosis in ACHD similar to other structural heart diseases. A systematic approach called sequential segmental analysis is recommended for delineating anatomy. Cardiovascular anatomy is defined in 3 segments in this examination protocol: visceral and atrial situs, atrioventricular concordance and the interrelation of great arteries. Subcostal, suprasternal, suprasternal views and modified parasternal views should be utilized liberally. A systematic approach with a standardized examination protocol and a report template allows a common language between clinics and prevents missed diagnosis of associated abnormalities (8).

Three-dimensional transthoracic echocardiography is a promising modality for delineation of complex anatomic abnormalities of the heart. This technique is especially advantageous in distorted chamber geometries in which 2D ejection fraction calculation methods are source of error. Dependence on operator experience and need for familiarity with the software of the specific device, higher cost, larger acoustic window required by bigger probes and need of multi-beat images with breath-hold for high frame-rate images are the main disadvantages (9).

Three-dimensional transesophageal echocardiography (3D-TEE) is particularly valuable during transcatheter occlusion of interatrial andinterventricular septal defects. Significant decrease in radiation exposure was observed with efficient use of 3D-transesophageal echocardiography (3D-TEE) in closure of interatrial communications. Limitations are less-pronounced compared to 3D-TTE (10). Fusion imaging with software-assisted superposition of 3D-echocardiography and fluoroscopy images on a single screen has been introduced recently and being tested in various different clinical trials (11).

Left and right ventricular strain imaging are the emerging modalities for assessment of contraction dynamics. Abnormalities in strain patterns often precede overt dysfunction.

Usefulness of strain imaging in several ACHD groups, including patients with bicuspid aortic valve, Tetralogy of Fallot (TOF) and operated TOF patients was previously shown (12,13,14). Strain imaging is effective in risk stratification of patients for closer follow-up or timing of transcatheter or surgical interventions. Limitations include need for high-quality 2D images, dependence on operator experience, significant intra-observer, inter-observer and cross-platform variability, potential errors in interpretation due to use of plug-ins developed for left ventricular strain imaging in assessment of right ventricular or left atrial mechanics and limitations associated with anatomical variations such as extensive right ventricle enlargement or univentricular hearts.

### Cardiac magnetic resonance imaging

**Figure 2:**
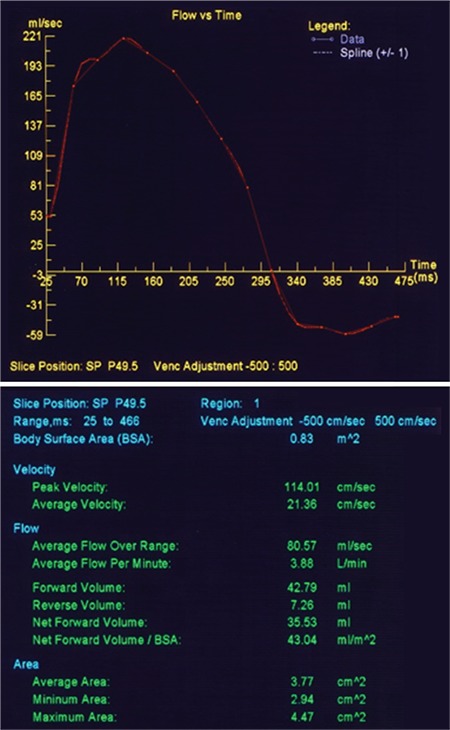
MRI quantification of pulmonary regurgitation. In this patient the regurgitation fraction was calculated as 17% by flow analysis.

Magnetic resonance imaging (MRI) demonstrates superior performance in volumetric calculations, 3D anatomy, wall characteristics and quantification of mass of ventricles and atria, shunts and shunt fractions, and regurgitant fractions of valvular incompetence and myocardial deformation measures. Magnetic resonance imaging is especially beneficial in assessments of the right heart including right ventricle, pulmonary valve and pulmonary artery. Magnetic resonance imaging is used for the right ventricle diastolic, systolic volume and ejection fraction measurements. The regurgitation fraction of pulmonary valve incompetence can be measured accurately by MRI (Figure 2). Tissue characterization allows fibrosis, scar, substance deposits and normal myocardium to be differentiated. Limitations include cost, need for interpreter experience, time-consuming nature of the technique, potential hazards in patients with rhythm devices and artifact-related decreases in image quality in patients with metallic prostheses despite the fact that many newer prosthetic heart valves are MRI compatible. Advent of MRI compatible pacemakers might resolve this problem and are recommended in patients with CHD. It is important that MRI compatibility of all the previously implanted devices in an ACHD patient under long-term follow-up assessed carefully (15).

### Computed tomography

Cardiac computerized tomography is more widely available and cheaper, application is less time-consuming and patient compliance is achieved easier compared to CMR. Nevertheless, due to concerns about ionizing radiation and intravenous contrast administration, we usually prefer CMR unless concurrent imaging for epicardial coronary arteries, pulmonary vascular tree and aorta, or other fistulae or shunts is planned (16).

### Cardiopulmonary exercise stress testing

Cardiopulmonary exercise testing (CPET) evolved to be used in exercise medicine but found wide application in ACHD in recent years. The test provides significant amount of data about hemodynamic response, conduction system and respiratory response to exercise and some parameters are still to be understood. The main advantage of this test would be the thorough information it provides, while the main disadvantage would be the expertise required for performance and interpretation of the test and limited availability. We utilize CPET liberally in our institution in guiding pharmacological, interventional and device therapy, as well as evaluation of the change in postprocedural progression of the diseases. Some examples to important applications of CPET in ACHD are as follows: patients with coarctation of the aorta with borderline clinical findings for reintervention, follow-up of patients with pulmonary hypertension and Eisenmenger syndrome, risk stratification and clinical decision-making in patients with repaired tetralogy of Fallot (TOF) (17,18,19). Applications of CPET in ACHD are still evolving.

## SELECTED CONGENITAL HEART DISEASES COMMONLY DISCOVERED DURING ADULTHOOD

### Atrial septal defect

Atrial septal defect is the most common form of congenital heart disease in the adult. The incidence is about 1-2%. It is not uncommon for an adult cardiologist to detect an ASD in a patient presenting with atypical chest pain or palpitations. Other presenting symptoms include exercise limitation, dyspnea and rarely right-sided heart failure, recurrent pulmonary infections or paradoxical emboli. Secundum-type ASD is the most common form and makes up more than two-third of the defects in the interatrial septum. 3D-TEE guidance and intracardiac echocardiography contributed to already-high procedural success with transcatheter closure, as well as postprocedural assessment of closure devices (20). A shunt of at least 1.5:1 calculated by cardiac catheterization, as well as dilation of the right heart chambers secondary to significant left to right shunt, were indications for closure before the development of advanced noninvasive imaging methods.

The main important question to be answered by a cardiologist in an adult patient with ASD today should be suitability for transcatheter closure. Echocardiography is the main modality in deciding for closure. There are several issues to be addressed in management of an ASD patient are as follows:

1. Presence of right ventricular failure and/or pulmonary hypertension: Enlargement in right heart chambers is an indicator of a significant ASD. Pulmonary artery systolic pressures should be assessed by echocardiography. Right heart catheterization should be performed, vascular resistance calculations should be made. Reversibility should be performed if significant pulmonary hypertension is detected. Eisenmenger physiology is a contraindication to percutaneous or surgical closure.

Concomittant Ebstein’s abnormality should be suspected in case of marked right atrial enlargement. More than 50% of patients with Ebstein’s abnormality have patent foramen ovale or ASD.

2. Anatomical properties of the defect: Is the defect secundum, primum or high-venosum? Transesophageal echocardiography is usually recommended and performed for unusual anatomy and poor echocardiographic window.

Superior or inferior sinus venosus defects may accompany partial abnormal pulmonary venous return. Pulmonary venous return abnormalities might be difficult to detect with transthoracic echocardiography.

3. Systolic or diastolic dysfunction of the left ventricle (secondary to ischemia, hypertension, valvular disease) might result in postprocedural complications after ASD closure. Flow redirected to right heart through ASD creates a relief for the chronic volume overload on the left ventricle, which has decreased compliance in some patients. Since increasing left ventricular stiffness and diastolic dysfunction is a common finding in the older adult, one should always be careful while proceeding with closure. This might result in reflection of the volume load back into pulmonary vasculature with resultant pulmonary edema in case of transcatheter occlusion.

4. Arrhythmias, especially atrial arrhythmias are a problem in patients with ASD. Atrial arrhythmias are more common in patients undergoing delayed transcatheter or surgical correction. Fifteen to twenty percent of patients experience some form of new-onset supraventricular tachyarrhythmia following correction (21). Antiarrhythmic medications or catheter ablation or surgical maze procedure in the same session with surgical correction might be preferred based on the clinical settings.

5. Paradoxical emboli: An ASD discovered in a patient presenting with paradoxical emboli prompts intervention regardless of the size, unless other contraindications exist.

### Indications for the defect interventional ASD closure in adults

*  Simple secundum ASD with significant shunt causing enlarged right heart chambers,

*  Sufficient rims,

*  Normal or slightly increased pulmonary artery pressure, without pulmonary vascular disease or Eisenmenger physiology.

***Strategies during interventional closure***

One of the most important implications of percutaneous closure method, specific for an adult patient with ASD might be the left ventricle diastolic dysfunction. Balloon occlusion test could be used in older patients with suspected diminished left ventricular compliance. The occlusion test could be done with the sizing balloons. Transient inflation of the balloon which blocks the left to right shunt of fifteen minutes duration is preferred method to reveal any hemodynamic disturbance. If there is an abnormal response to balloon occlusion, closure with a fenestrated device could be considered. Another strategy is left ventricle training with drugs to treat the diminished left ventricle compliance for a few weeks. The closure could be possible after this treatment period (22). However, the concept of adverse outcome related with increased left ventricular end diastolic pressures following a transcatheter ASD closure is controversial (23).

### Patent ductus arteriosus

artery to obliterate after birth. Most of the PDAs are diagnosed and closed during childhood. Percutaneous closure again attracts interventional cardiologists.

Mild continuous murmur is the main presenting clinical finding in many patients with a PDA. This defect may incidentally be diagnosed during TTE performed for various other reasons. PDA is usually detected in basal sections of parasternal short axis window or distal half of the aortic arch in suprasternal window. Closure of a silent PDA is an ongoing debate today. Some authors suggest that even silent shunts might alter long-term survival and prognosis patterns during follow-up (24). Most patients may be closed by interventional methods, surgery is rarely needed and it is mainly reserved for large defects. The main consequences of a significant PDA are volume overload with left ventricular enlargement and dysfunction.

Eisenmenger syndrome may develop at large PDA’s missed during childhood period. Correction is contraindicated in this instance. Endarteritis became less common in recent years. Aneurysm formation is a late complication due to endarteritis.

### Coarctation of the aorta

Aortic coarctation (ACo) is defined as focal constriction in descending aorta just distal to aortic attachment of ligamentum arteriosum. There are anatomical variations in extent of stenosis caused by and segments involved. The condition is frequently diagnosed during childhood but the most common clinical presentation in an adult is upper extremity hypertension. It is important to note that ACo is a systemic disease and should not be perceived as a simply mechanical obstruction. Hypertension and the other related vascular consequences might persist or recur during follow-up of patients who undergo correction. Ambulatory blood pressure monitoring (ABPM) and exercise stress testing might be used in follow-up. Recurrence might manifest as a hypertensive response to exercise stress testing or findings in ABPM. Hypertension due to aortic coarctation should be treated aggressively. Noninvasive and invasive imaging methods should be used for recognition of recoarctation (19). Hypertension due to aortic coarctation should be treated aggressively.

**Figure 3:**
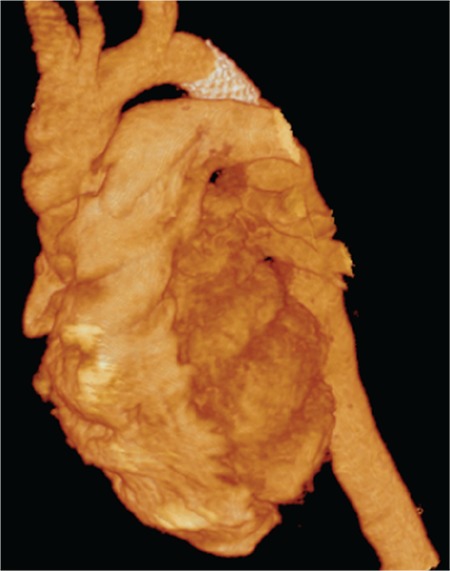
Three-dimensional imaging of aorta in a patient with previous bare stent implantation for aortic coarctation.

Although echocardiography is essential as the initial diagnostic modality, definitive diagnosis necessitates computerized tomography/magnetic resonance angiography and/or invasive angiography. Computerized tomography-angiography is the preferred modality unless there is a contraindication (Figure 3). Upper extremity hypertension, a peak gradient more than 20 mmHg in patients with or without hypertension, more than 50% of stenosis compared to the aortic diameter at the level of diaphragm indicate a significant coarctation, warranting correction. In patients presenting with recoarctation, diagnostic criteria and intervention indications are the same (6).

Transcatheter stent implantation, preferably covered stent, is shown to be safe and efficient in treatment of this disease and was used successfully even in near-atretic coarctation cases. Surgical treatment is reserved for patients who are not eligible for transcatheter intervention due to anatomical or technical reasons. There are a wide variety of surgical techniques for correction of aortic coarctation but the 3 main techniques are: end to end anastomosis, interposition graft and jump graft (25)

## SELECTED PREVIOUSLY OPERATED CONGENITAL HEART DEFECTS

### Fontan circulation

**Figure 4:**
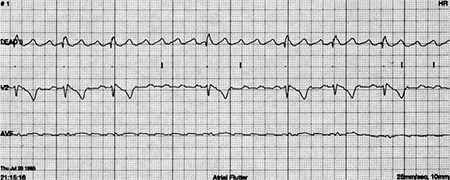
Holter recording in a patient with previous atriopulmoanry Fontan. There is atrial tachycardia with slow ventricular rhythm.

A subset of patients with previously corrected CHD has a single active pump unit receiving pulmonary venous flow and directing flow to systemic circulation. Fontan procedure, or cavo-pulmonary circulation is surgical bypass of the right ventricle to divert systemic venous blood directly to pulmonary circulation. The procedure, introduced in late 1960s, was suggested in an effort to provide cure for atretic tricuspid valve (26). The classical procedure is called atriopulmonary Fontan, which is associated with problems such as loss of sinus rhythm, atrial tachyarrhythmias, protein losing enteropathy (Figure 4). Several modifications have been defined to overcome longterm complications. The subtype called extracardiac Fontan seems to provide better results compared to atriopulmonary and lateral tunnel subtypes (27). The clinical presentations that mimic more common heart diseases (i.e. systemic venous congestion due to right heart failure) should be managed with caution, since response to different therapeutic interventions might result in unexpected complications (28).

Rhythm disturbances are not rare and mainly consist of atrial arrhythmias and atrial fibrillation. Atrial fibrillation is a major problem with atriopulmonary Fontan patients and increases with duration of follow-up (29). It is suggested that effect of atrial contraction might have beneficial effects in Fontan circulation and sinus rhythm should be maintained if possible. Impact of arrhythmias on morbidity and mortality is not clear, though. Other common etiologies such as ischemia should always be kept in mind in case of arrhythmia in an adult patient. Radiofrequency ablation might be performed albeit with hight recurrence rates. Ventricular arrhythmias and sudden cardiac death were also reported (30).

**Figure 5:**
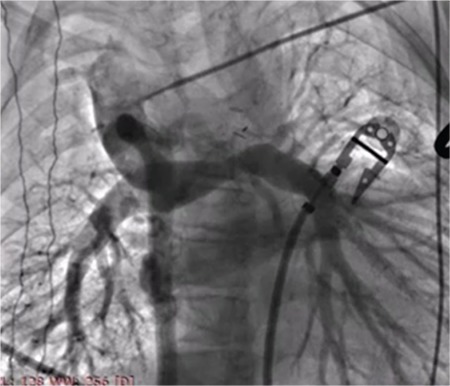
Kawashima operation in a patient with single ventricle physiology. There is narrowing at the proximal part of the left pulmonary artery.

Systemic congestion and protein-losing enteropathy are other complications. Lack of suction effect from active relaxation of the right ventricle and augmentation of the pulmonary venous flow is held responsible for this (Figure 5). Impedance of this iatrogenic neoportal system becomes the main determinant of the cardiac output in this case. Therapeutic strategies are developed to overcome this main obstacle to forward circulation in failing Fontan (31). Many patients with single ventricle physiology underwent cavo-pulmonary anastomosis may need cardiac transplantation at long-term follow-up.

### Corrected TOF

Tetralogy of Fallot is the most common form of cyanotic congenital heart disease. Most of the patients have been diagnosed and surgically repaired during infancy period. While the palliative approach consists of Blalock-Taussig shunt, Waterston and Potts anastomoses may be needed in patients with anatomy unsuitable for this shunt. The rationale underlying each of these shunts is directing blood from either left subclavian artery-to left main pulmonary artery or ascending aorta-to-right main pulmonary, or descending aortic-to left main pulmonary artery, respectively. These shunts are preferred either until the total correction is performed or when the patient is unamenable for a therapeutic surgical intervention. Definitive treatment of TOF consists of right ventricle outflow tract (RVOT) repair and VSD closure. Right ventricular outflow tract repair could be performed either with simple infundibular resection or transannular patch might be required in patients with more severe outflow obstruction.

In patients with a definitive surgical correction there are several issues which demand special attention: pulmonary regurgitation, residual or recurrent RVOT obstruction, and atrial and ventricular arrhythmias.

***Pulmonary regurgitation***

**Figure 6:**
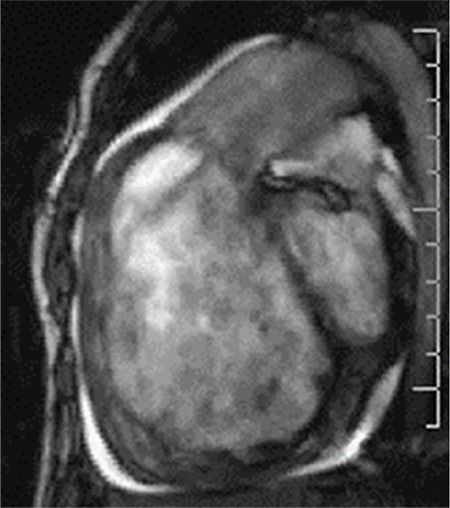
Magnetic resonance imaging of the right ventricle and pulmonary regurgitation in a patient with tetralogy repair. The right ventricle is enlarged and regurgitant jet (black arrow) is seen.

Pulmonary regurgitation (PR) is more severe and more common in patients with transannular patch repair. Pulmonary regurgitation was found associated with fatal and nonfatal arrhythmias compared to either significant tricuspid regurgitation or pulmonary hypertension (32). It is very important that detailed assessments should be performed in patients with TOF, and accompanying peripheral pulmonary stenosis should be investigated. Timing of pulmonary valve replacement in patients with significant PR is an important question. Right ventricular enlargement determined by cardiac MRI (Figure 6), significant decrease in maximal oxygen consumption in CPET (VO2 max), progressive QRS prolongation, progressive symptoms, left or right ventricular systolic dysfunction are proposed indications. Patients respond to pulmonary valve replacement (PVR) excellently on clinical basis, although a meta-analysis concluded that PVR was not associated with significant recovery in right ventricular ejection fraction (33,34).

***Recurrent right ventricle outflow tract obstruction***

Increased right ventricular systolic pressures by echocardiography should always warrant cardiac catheterization. Site of stenosis might be at the level of infundibular, valvar or any other distal site as well. Percutaneous or surgical interventions for distal stenoses might be the treatment of choice. The obstruction should be treated in case of right ventricle systolic pressure exceeding 2/3 of left ventricle systolic pressure.

***Arrhyhthmias***

**Figure 7:**
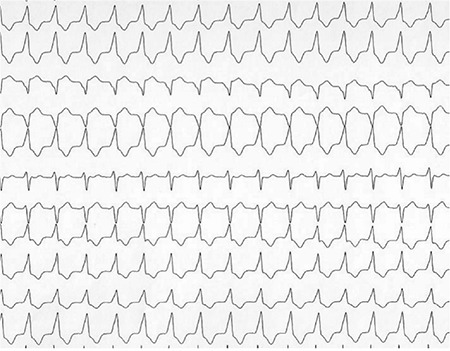
Wide QRS tachycardia in a patient with previous repair of tetralogy. The patient underwent a cardiac electrophysiologic study and an implantable cardioverter defibrillator was implanted.

Wide-variety of arrhythmias, ranging from atrial fibrillation to supraventricular tachycardias to ventricular tachycardias may occur during follow-up (Figure 7). Arrhythmias might also be associated with deterioration of left and/or right ventricular systolic function, exercise intolerance, syncope or sudden cardiac death might implicate an underlying arrhythmogenic etiology. Bundle branch block is also frequent in these patients. QRS prolongation is an indicator of unfavorable outcome and precludes intervention for valvular disease.

Of note, an untouched adult TOF patient with an incidental late presentation is still a good candidate for surgical correction provided that there is no severe irreversible pulmonary vascular disease or severe right ventricular dysfunction.

### Senning, Mustart Operations (atrial switch) and Jatene (arterial switch) procedure

Patients with previous arterial switch operations, performed for the relatively common congenital heart disease called transposition of great arteries (d-TGA) is another group worth discussing.

There are 2 types of TGA. The first type is called corrected (c-TGA or L-TGA) with discordant atrioventricular and ventriculoarterial association, and these patients might reach adult age without an apparent problem unless there is a concomitant shunt, which is a frequent finding in these patients. The second type is called d-TGA and this defect is incompatible with life unless there is a concomitant left-right shunt. Surgical correction for this disease underwent significant evolution to take its final form, Jatene procedure, which consists of reimplantation of great arteries to their consequent ventricles and reimplantation of coronary arteries to neo-aorta.

The previously mentioned segmental analysis is vital in delineating anatomy in patients with transposition. Venoatrial, atrioventricular and ventriculoarterial associations should be carefully assessed by proper imaging techniques and interpreted by experienced imagers.

Mustard and Senning procedures formed the basis of surgical correction of d-TGA for about 2 decades and many of these patients are now in their adulthood. These operations are based on the same technique of forming a baffle, directing blood from systemic veins to left ventricle and from pulmonary veins to right ventricle. The only difference is that while Mustard makes use of pericardial tissue, atrial tissue is used in Senning procedure. Although survival after Mustard and Senning procedures improved in the last 2 decades, Jatene procedure seems to provide better outcomes (80% with atrial switch procedures versus 100% survival with Jatene procedure over 20 years) (35).

***Main problems related with these operations are***

**Figure 8:**
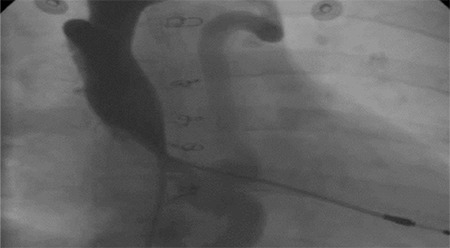
Baffle obstruction over vena cava superior connection in a patient with previous Senning operation (atrial switch) (white arrow).

 1.Baffle leak or obstruction: Baffle leaks are restrictive most of the time and become clinically relevant when either the defect is large or is proximal to a baffle obstruction with resultant right-to-left shunting (Figure 8).

2. Arrhythmia: Atrial manipulation during these procedures predispose to nodal disease and atrial arrhythmias. Many patients will need a pacemaker. Catheter ablation, although technically demanding might be an effective option.

 3. Outflow obstruction might be observed due to relatively thick left ventricular wall in the subpulmonary region. Subvalvular membranes might or might not accompany.

4. Right ventricular dysfunction: Right ventricular dysfunction is an important cause of morbidity in patients with TGA and there should be an active ongoing surveillance for early symptoms and signs (36).

## SPECIAL CONSIDERATIONS in ACHD

### Sudden cardiac death

Sudden Cardiac Death (SCD) is a catastrophic consequence of ACHD. Congenital heart disease is demonstrated as the cause of SCD in %0.02 of patients in a recent autopsy study (37). Recent developments in telemetric monitorization technology enabled early detection of arrhythmic episodes which would otherwise lead to SCD (38).

Sudden cardiac death has two main implications in adult congenital heart disease patients. First and maybe the most important one would be risk stratification and primary prevention. Second one would be the technical challenges related with implantable cardioverter defibrillator (ICD) implantation related with insertion of leads in surgically manipulated hearts.

QRS duration and dispersion as well as temporal changes in these parameters, left ventricle ejection fraction and subpulmonary ventricular functions were predictors of sudden cardiac death in ACHD patients. QRS prolongation was in the form of nonspecific conduction delay in patients with preserved ventricular functions and in the form of bundle branch block in ACHD patients. QRS prolongation demonstrated a more rapid progression in patients with systemic or subpulmonary ventricular systolic dysfunction (39).

Almost one-third of ACHD patients required reintervention following implantation of a rhythm device with defibrillator capability. Forty-percent of the ICD shocks delivered in these patients were classified as inappropriate (40).

### Infective endocarditis

IE is a major concern in ACHD. It is particularly important in patients with residual or untreated left to right shunts. The latest European Society of Cardiology on IE are far more loose compared with the previous guidelines with regard to antibiotic prophylaxis. The patient and disease related factors that necessitate prophylaxis according to these guidelines are:

1. The first 6 months following surgical or transcatheter treatment of intracardiac shunts.

2. Any residual leak or valvular leak following transcatheter closure of shunts.

3. Presence of any type of prosthetic heart valve.

4. Any type of cyanotic congenital heart disease.

5. Previous history of IE.

**The procedure related factors are as follows:**

1. Dental procedures involving gingiva, periapical region or that results in loss of continuity of the oral mucosa will require prophylaxis with proper antibiotic regimen,

2. Surgical interventions involving respiratory system, gastrointestinal system, genitourinary system, musculoskeletal system or skin and already require prophylaxis by nature would require IE prophylaxis to cover potential microorganisms. Prophylaxis is required for any type of vascular or cardiac prostheses. Prophylaxis is not recommended for procedures that don’t fit in any of these categories (41).

### Woman health and pregnancy

***Reproductive health and contraception***

It is shown that menarche is significantly delayed in patients with CHD. Primary, secondary amenorrhea and menstrual cycle irregularities were more common in women with CHD. The authors emphasized the role of functional hypothalamic amenorrhea, which could be exacerbated with stress response, hemodynamic disturbances and cachexia due to heart failure (42). Menstrual cycle irregularities were more common among persistently cyanotic TOF patients previously treated with palliative surgery (43). Various hormone-based treatments are available for uterine bleeding and menstrual cycle irregularities. Preparations containing estrogen are not preferred in patients with high thrombotic risk (44). Many ACHDs carry increased risk of thrombosis and some hormonal treatments may be harmful.

Women in reproductive age should be counseled about their disease and the associated pregnancy related risk by a multidisciplinary team. Patients on potentially teratogenic cardiovascular drugs should be warned against risks of pregnancy.

Non-hormonal intrauterine devices are the first choice for contraception. When hormone-based methods are preferred, preparations containing estrogen are not recommended due to same reasons discussed above (45).

***Pregnancy, delivery and lactation***

Pregnancy poses a significant risk for the fetus and the mother in certain forms of ACHD while others could be well-tolerated with outcomes comparable to general population. Different risk models have been proposed for risk stratification (46,47). European Society of Cardiology recommends use of modified World Health Organization (WHO) risk classification (48,49). The conditions pregnancy is contraindicated and designated WHO class IV are: pulmonary arterial hypertension, severe left ventricle systolic dysfunction with a left ventricular ejection fraction of  less than 30% and/or New York Heart Association Class (NYHA) III-IV heart failure, severe mitral or aortic stenosis, native severe coarctation of the aorta, Marfan syndrome with aortic diameter of greater than 45 mm or bicuspid aortic valve with an aortic diameter of greater than 50 mm. Other risk classes are summarized in Table 1.

***Cardiovascular drugs during pregnancy in patients with ACHD:*** Pharmacotherapy should be tailored for pregnancy. Classes of cardiovascular drugs used in ACHD patients are: anticoagulants, antiarrhythmic agents, antihypertensive agents and heart failure medications. Unfortunately, hazardous effects of most of these drugs in pregnancy are well known.

Angiotensin converting enzyme inhibitors and angiotensin receptor blockers are associated with several congenital abnormalities along with oligohydramnios, intrauterine growth retardation, prematurity and renal failure (50). Beta blockers are associated with fetal bradycardia, low placental weight and hypoglycemia. Digoxin is beneficial for arrhythmias in fetus and mother without teratogenic effects. Diuretics are helpful in management of heart failure, but are associated with reduced utero-placental perfusion, fetal hypoglycemia, hyponatremia and hypokalemia. Spironolactone causes oral clefts in first trimester of pregnancy and it has antiandrogenic effects as well. Heparin is a prominent anticoagulant which has no reported effects on fetus (51). Warfarin is a vitamin K antagonist which is used as an anticoagulant. It is related with a set of abnormalities termed Coumarin embryopathy, as well as risk of fetal bleeding. The critical period for warfarin embryopathy is 6-12 weeks and 36th week to delivery. Latest guidelines recommend that oral anticoagulants could be used safely except these critical periods. However, continuation of the vitamin K antagonist might be preferred in selected patients with daily maintenance doses of warfarin less than 5 mg between 6^th^ and 12^th^ weeks. Discontinuation or continuation of vitamin K antagonist should be decided on an individual basis, weighing the risks and benefits. Selected drug classes used during pregnancy in ACHD patients are summarized in Table 2 (49).

***Menopause and hormone replacement therapy***

As discussed in the previous section, primary and secondary ovarian failure and early menopause seem to be more common in certain ACHD subgroups (42). Hormonal replacement therapy has been found associated with cerebrovascular disease and venous thromboembolism (7). Although certain subsets of ACHD patients seem to be safe with respect to thrombotic risk, most gynecologists prefer to stay on the safe side in prescribing hormonal therapies.

### Male reproductive health and erectile dysfunction

Erectile dysfunction is frequent among ACHD patients. Severity of erectile dysfunction does not seem to correlate with the severity of ACHD. Nitrate use should be questioned when phosphodiesterase inhibitors are considered for treatment of erectile dysfunction.

### Psychosocial issues

ACHD patients face many problems related to their care in many levels. These patients are usually overwhelmed by the frequent physician visits and interventions. Majority have complicated medical records with complicated imaging, intervention and surgery notes (49). Their medical records are completely or partially lost or damaged in some cases. Adult cardiologists often lack expertise for management of ACHD (52). Studies demonstrated that approximately half of the adolescents reaching adulthood thought that the information they receive from caregivers is not enough for them. Many had concerns regarding adult life such as employment, family planning and the long-term course of their disease (53). It is apparent that preparation for the multiple domains of adult life is as vital as planning of the health care during adulthood and this transition should be supported by a multidisciplinary team.

Denial reactions are common in terminal stage patients with CHD. Management of these patients might be challenging for an unexperienced physician. Studies showed that denial is more common in ACHD patients and had impact on adherence to medical therapy (54).

NYHA class is shown to be a significant predictor of QOL in these patients (55). Mood and anxiety disorders are the leading psychiatric disorders in this age group. Alcohol and nicotine abuse significantly affect QOL in this patient group (56).

***Palliative Care***

Palliative care and end-of-life discussions constitute an extremely important aspect of care when currently available therapy options are already exercised. Symptom relief and treatment of the mood disorders, as well as thorough end-of-life care discussions with involvement of the specialists in the field is very important (57). Unfortunately, only a minority of the patients with terminal ACHD are shown to benefit from specialized palliative care and similarly a small proportion were involved in attentive end-of-life discussions, previously (58).

## Figures and Tables

**Table 1:**
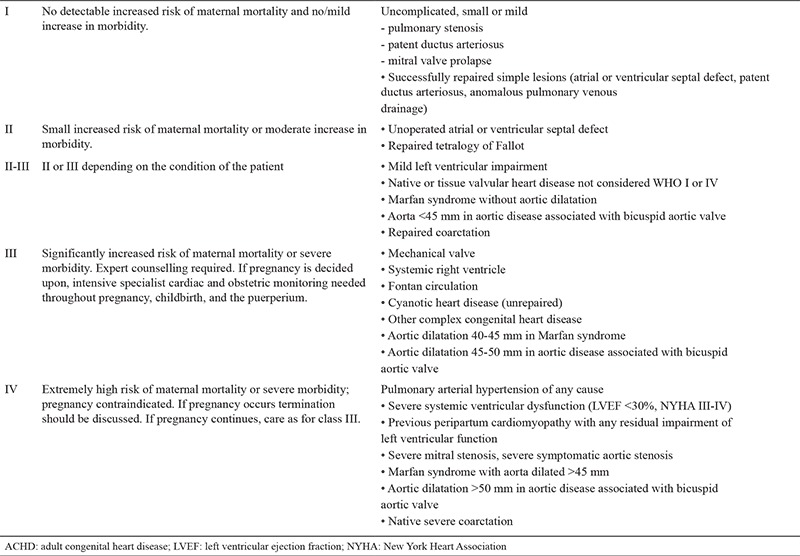
WHO risk stratification of ACHD in pregnancy (The table is modified to cover the diseases relevant to text)

**Table 2:**
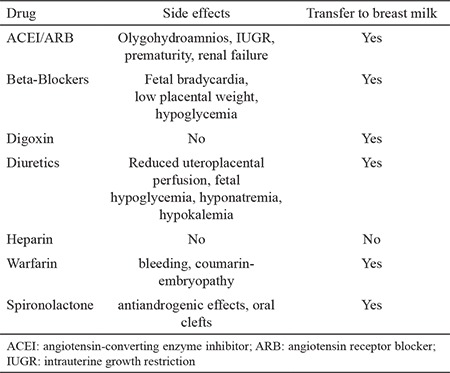
Common used drugs and their effects on fetus and newborn baby (The table is modified to cover the diseases relevant to text) (49)

**Figure 1:**
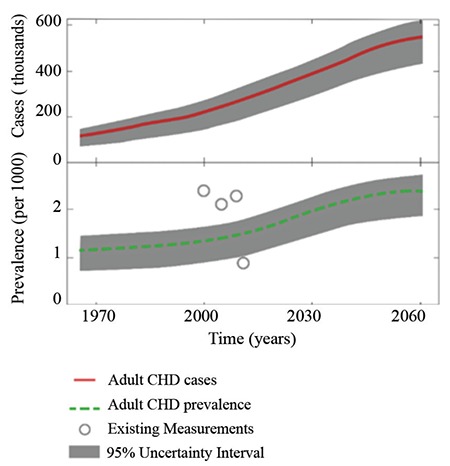
Adapted from (2)]: Projected growth of the adult congenital heart disease population in the United States to the year 2050. Upper graph with the red trajectory shows the estimated number of cases, while the lower graph with the dotted green trajectory shows the estimated prevalence. Case numbers and prevalence shows a hyperbolic increase with a plateau in the 2050s.

## References

[1] Kelleher AA (2012). Adult congenital heart disease (grown-up congenital heart disease). Continuing Education in Anaesthesia Critical Care and Pain.

[2] Benziger CP, Stout K, Zaragoza-Macias E, Bertozzi-Villa A, Flaxman AD (2015). Projected growth of the adult congenital heart disease population in the United States to 2050: an integrative systems modeling approach. Popul Health Metr.

[3] Macmahon B, McKeown T, Record RG (1953). The incidence and life expectation of children with congenital heart disease. Br Heart J.

[4] Wray J, Frigiola A, Bull (ACoRN) (2013). Loss to specialist follow-up in congenital heart disease; out of sight, out of mind. Heart.

[5] Marmagkiolis K, Arzamendi D, Goktekin O, Cilingiroglu M (2016). Structural heart interventions training in Europe. Int J Cardiol.

[6] Baumgartner H, Bonhoeffer P, De NM, de F, Deanfield JE, Galie N, et al (2010). ESC Guidelines for the management of grown-up congenital heart disease (new version 2010). Eur Heart J.

[7] Bhatt AB, Foster E, Kuehl K, Alpert J, Brabeck S, Crumb S, et al (2015). Congenital heart disease in the older adult: a scientific statement from the American Heart Association. Circulation.

[8] Anderson RH, Becker AE, Freedom RM, Macartney FJ, Quero-Jimenez M, Shinebourne EA, et al (1984). Sequential segmental analysis of congenital heart disease. Pediatr Cardiol.

[9] Bleich S, Nanda NC, Hage FG (2013). The incremental value of three-dimensional transthoracic echocardiography in adult congenital heart disease. Echocardiography.

[10] Balzer J, van S, Rassaf T, Böring YC, Franke A, Lang RM, et al (2010). Feasibility, safety, and efficacy of real-time three-dimensional transoesophageal echocardiography for guiding device closure of interatrial communications: initial clinical experience and impact on radiation exposure. Eur J Echocardiogr.

[11] Jone PN, Ross MM, Bracken JA, Mulvahill MJ, Di MV, Fagan TE (2016). Feasibility and Safety of Using a Fused Echocardiography/Fluoroscopy Imaging System in Patients with Congenital Heart Disease. J Am Soc Echocardiogr.

[12] Santarpia G, Scognamiglio G, Di Salvo G, D’Alto M, Sarubbi B, Romeo E, et al (2012). Aortic and left ventricular remodeling in patients with bicuspid aortic valve without significant valvular dysfunction: a prospective study. Int J Cardiol.

[13] Diller GP, Kempny A, Liodakis E, Alonso-Gonzalez R, Inuzuka R, Uebing A, et al (2012). Left ventricular longitudinal function predicts life-threatening ventricular arrhythmia and death in adults with repaired tetralogy of fallot. Circulation.

[14] van der Hulst AE, Delgado V, Holman ER, Kroft LJ, de Roos A, Hazekamp MG, et al (2010). Relation of left ventricular twist and global strain with right ventricular dysfunction in patients after operative “correction” of tetralogy of fallot. Am J Cardiol.

[15] Bonnichsen C, Ammash N (2016). Choosing Between MRI and CT Imaging in the Adult with Congenital Heart Disease. Curr Cardiol Rep.

[16] Han BK, Rigsby CK, Hlavacek A, Leipsic J, Nicol ED, Siegel MJ, et al (2015). Computed Tomography Imaging in Patients with Congenital Heart Disease Part I: Rationale and Utility. An Expert Consensus Document of the Society of Cardiovascular Computed Tomography (SCCT): Endorsed by the Society of Pediatric Radiology (SPR) and the North American Society of Cardiac Imaging (NASCI). J Cardiovasc Comput Tomogr.

[17] Buys R, Van De Bruaene A, Müller J, Hager A, Khambadkone S, Giardini A, et al (2013). Usefulness of cardiopulmonary exercise testing to predict the development of arterial hypertension in adult patients with repaired isolated coarctation of the aorta. Int J Cardiol.

[18] Yang-Ting S, Aboulhosn J, Sun XG, Child JS, Sietsema KE (2011). Effects of pulmonary vasodilator therapy on ventilatory efficiency during exercise in adults with Eisenmenger syndrome. Congenit Heart Dis.

[19] Babu-Narayan SV, Diller GP, Gheta RR, Bastin AJ, Karonis T, Li W, et al (2014). Clinical outcomes of surgical pulmonary valve replacement after repair of tetralogy of Fallot and potential prognostic value of preoperative cardiopulmonary exercise testing. Circulation.

[20] Bhaya M, Mutluer FO, Mahan E, Mahan L, Hsiung MC, Yin WH, et al (2013). Live/Real time three-dimensional transesophageal echocardiography in percutaneous closure of atrial septal defects. Echocardiography.

[21] Wang JK, Chiu SN, Lin MT, Chen CA, Lu CW, Wu MH (2017). Mid-to-long-term follow-up results of transcatheter closure of atrial septal defect in patients older than 40 years. Heart Vessels.

[22] Schubert S, Peters B, Abdul-Khaliq H, Nagdyman N, Lange PE, Ewert P (2005). Left ventricular conditioning in the elderly patient to prevent congestive heart failure after transcatheter closure of atrial septal defect. Catheter Cardiovasc Interv.

[23] Ermis P, Franklin W, Mulukutla V, Parekh D, Ing F (2015). Left ventricular hemodynamic changes and clinical outcomes after transcatheter atrial septal defect closure in adults. Congenit Heart Dis.

[24] Gologorsky E, Giquel J, Gologorsky A (2011). A silent patent ductus arteriosus: a culprit or an innocent bystander? World J Pediatr Congenit Heart Surg 2011;2:129-32. 2011;2:129-32..

[25] Monro J (2005). The changing state of surgery for adult congenital heart disease. Heart.

[26] Fontan F, Baudet E (1971). Surgical repair of tricuspid atresia. Thorax.

[27] Lardo AC, Webber SA, Friehs I, del PJ, Cape EG (1999). Fluid dynamic comparison of intra-atrial and extracardiac total cavopulmonary connections. J Thorac Cardiovasc Surg.

[28] Gewillig M, Brown SC (2016). The Fontan circulation after 45 years: update in physiology. Heart.

[29] Paul T, Ziemer G, Luhmer L, Bertram H, Hecker H, Kallfelz HC (1998). Early and late atrial dysrhythmias after modified Fontan operation. Pediatr Med Chir.

[30] Fuchigami T, Nagashima M, Hiramatsu T, Matsumura G, Tateishi M, Masuda N, et al (2017). Long-term follow-up of Fontan completion in adults and adolescents. J Card Surg.

[31] Gewillig M, Brown SC (2016). The Fontan circulation after 45 years: update in physiology. Heart.

[32] Gatzoulis MA, Balaji S, Webber SA, Siu SC, Hokanson JS, Poile C, et al (2000). Risk factors for arrhythmia and sudden cardiac death late after repair of tetralogy of Fallot: a multicentre study. Lancet.

[33] Ferraz PE, Sá MP, Santos CA, Esmeraldo IM, de RR, de AM, et al (2013). Pulmonary valve replacement after operative repair of tetralogy of Fallot: meta-analysis and meta-regression of 3,118 patients from 48 studies. J Am Coll Cardiol.

[34] van Straten A, Vliegen HW, Hazekamp MG, Bax JJ, Schoof PH, Ottenkamp J, et al (2004). Right ventricular function after pulmonary valve replacement in patients with tetralogy of Fallot. Radiology.

[35] Raissadati A, Nieminen H, Sairanen H, Jokinen E (2017). Outcomes after the Mustard, Senning and arterial switch operation for treatment of transposition of the great arteries in Finland: a nationwide 4-decade perspective. Eur J Cardiothorac Surg.

[36] Popelova J (2008). Transposition of Great Arteries. In: Popelova J, Oechslin E, Kaemmerer H, St John Sutton MG, editors.Congenital Heart Disease in Adults.

[37] Hamilton LE, Lew EO, Matshes EW (2011). “Grown-up” congenital heart disease and sudden death in a medical examiner’s population. J Forensic Sci.

[38] Nagel B, Janousek J, Koestenberger M, Maier R, Sauseng W, Strenger V, et al (2014). Remote monitoring leads to early recognition and treatment of critical arrhythmias in adults after atrial switch operation for transposition of the great arteries. Circ J.

[39] Koyak Z, de JR, Bouma BJ, Zwinderman AH, Silversides CK, Oechslin EN, et al (2017). Sudden cardiac death in adult congenital heart disease: can the unpredictable be foreseen?. Europace.

[40] Yap SC, Roos-Hesselink JW, Hoendermis ES, Budts W, Vliegen HW, Mulder BJ, et al (2007). Outcome of implantable cardioverter defibrillators in adults with congenital heart disease: a multi-centre study. Eur Heart J.

[41] Habib G, Lancellotti P, Antunes MJ, Bongiorni MG, Casalta JP, Del F, et al (2016). 2015 ESC Guidelines for the management of infective endocarditis. The Task Force for the Management of Infective Endocarditis of the European Society of Cardiology (ESC)]. G Ital Cardiol (Rome).

[42] Drenthen W, Hoendermis ES, Moons P, Heida KY, Roos-Hesselink JW, Mulder BJ, et al (2008). Menstrual cycle and its disorders in women with congenital heart disease. Congenit Heart Dis.

[43] Canobbio MM, Mair DD, Rapkin AJ, Perloff JK, George BL (1990). Menstrual patterns in females after the Fontan repair. Am J Cardiol.

[44] Canobbio MM, Perloff JK, Rapkin AJ (2005). Gynecological health of females with congenital heart disease. Int J Cardiol.

[45] Gialeraki A, Valsami S, Pittaras T, Panayiotakopoulos G, Politou M (2016). Oral Contraceptives and HRT Risk of Thrombosis. Clin Appl Thromb Hemost.

[46] Siu SC, Sermer M, Colman JM, Alvarez AN, Mercier LA, Morton BC, et al (2001). Prospective multicenter study of pregnancy outcomes in women with heart disease. Circulation.

[47] Drenthen W, Boersma E, Balci A, Moons P, Roos-Hesselink JW, Mulder BJ, et al (2010). Predictors of pregnancy complications in women with congenital heart disease. Eur Heart J.

[48] Thorne S, MacGregor A, Nelson-Piercy C (2006). Risks of contraception and pregnancy in heart disease. Heart.

[49] Regitz-Zagrosek V, Blomstrom C, Borghi C, et al, European Society of Gynecology (ESG), Association for European Paediatric Cardiology (AEPC), German Society for Gender Medicine (DGesGM) Regitz-Zagrosek V, Blomstrom Lundqvist C, Borghi C, et al (2011). ESC Guidelines on the management of cardiovascular diseases during pregnancy: the Task Force on the Management of Cardiovascular Diseases during Pregnancy of the European Society of Cardiology (ESC). Eur Heart J.

[50] Elkayam U (2005). Pregnancy and cardiovascular disease. In: Zipes DP, editor. Braunwald’s Heart Disease: A Textbook of Cardiovascular Medicine. 7th ed.

[51] Bonow RO, Carabello BA, Chatterjee K, et al, American College of Cardiology, American Heart Association Task Force on Practice Guidelines (Writing Committee to revise the 1998 guidelines for the management of patients with valvular heart disease), Society of Cardiovascular Anesthesiologists (2006). ACC/AHA 2006 guidelines for the management of patients with valvular heart disease: a report of the American College of Cardiology/American Heart Association Task Force on Practice Guidelines (writing Committee to Revise the 1998 guidelines for the management of patients with valvular heart disease) developed in collaboration with the Society of Cardiovascular Anesthesiologists endorsed by the Society for Cardiovascular Angiography and Interventions and the Society of Thoracic Surgeons. J Am Coll Cardiol.

[52] Popelova J (2008). Psychosocial issues. In: Popelova J, Oeschslin E, Kaemmerer H, Sutton MG, editors. Congenital Heart Disease in AdultsCongenital Heart Disease in Adults.

[53] Lopez KN, Karlsten M, Bonaduce F, King J, Salciccioli K, Jiang A, et al (2015). Understanding Age-based Transition Needs: Perspectives from Adolescents and Adults with Congenital Heart Disease. Congenit Heart Dis.

[54] White KS, Pardue C, Ludbrook P, Sodhi S, Esmaeeli A, Cedars A (2016). Cardiac Denial and Psychological Predictors of Cardiac Care Adherence in Adults With Congenital Heart Disease. Behav Modif.

[55] Apers S, Kovacs AH, Luyckx K, Thomet C, Budts W, Enomoto J, et al (2016). Quality of Life of Adults With Congenital Heart Disease in 15 Countries: Evaluating Country-Specific Characteristics. J Am Coll Cardiol.

[56] Westhoff-Bleck M, Briest J, Fraccarollo D, Hilfiker-Kleiner D, Winter L, Maske U, et al (2016). Mental disorders in adults with congenital heart disease: Unmet needs and impact on quality of life. J Affect Disord.

[57] LeMond L, Mai T, Broberg CS, Muralidaran A, Burchill LJ (2015). Heart Failure in Adult Congenital Heart Disease: Nonpharmacologic Treatment Strategies. Cardiol Clin.

[58] Tobler D, Greutmann M, Colman JM, Greutmann-Yantiri M, Librach LS, Kovacs AH (2012). End-of-life care in hospitalized adults with complex congenital heart disease: care delayed, care denied. Palliat Med.

